# Erosion of Inflatable Penile Prosthesis with Prolonged Foley Catheterization in the COVID-19 Era

**DOI:** 10.1016/j.esxm.2021.100371

**Published:** 2021-05-30

**Authors:** Sravan Panuganti, Sohail Dhanji, Run Wang

**Affiliations:** 1 Department of Urological Surgery, Rowan University School of Osteopathic Medicine, Stratford, NJ, USA; 2 Department of Urology, UT-Houston Medical School, Houston, TX, USA

The COVID-19 pandemic has changed the lives of many in the past year. As of writing this article, the virus has claimed over half a million American lives and has infected millions more.^1^ It has affected many people regardless of age, gender, race, religion, or medical history. We have noticed a unique sequence of events in urology patients with a prior history of inflatable penis prothesis implantation who have gotten critically ill from the Severe acute respiratory syndrome coronavirus 2.

We have encountered 3 patients with a very similar history in the past year. They were all men aged 57-72 years old who had a functioning inflatable penile prothesis (IPP) for many years (3-13) and were intubated for a prolonged period of time (2-4 wk) after suffering respiratory distress from the Severe acute respiratory syndrome coronavirus 2. During this time, they all had a prolonged urethral Foley catherization for urinary drainage while in the intensive care unit. They were all subsequently found to have urethral erosion of a penile implant cylinder which was not present prior to hospitalization. Two patients underwent explantation of their IPP during their hospital stay and one presented to our outpatient office 2 months after discharge with the complaint of urethral cylinder erosion and underwent subsequent explantation.

Urethral catheterization is commonly used in the intensive care unit and spinal cord injury patients due to their convenience and efficacy. The friction and inflammation created by prolonged transurethral catheterization can be disastrous for IPPs by increasing the likelihood of infection and/or device erosion. In fact, Steidle and Mulcahy found that five out of their nine patients (55%) with IPPs who had an indwelling or intermittent transurethral catheterization were eventually found to have erosion of their IPP.^2^ In addition, indwelling transurethral catheters also confer a higher risk of urinary tract infection. Han *et al.* found that suprapubic tube placement conferred a statistically significantly lower risk of urinary tract infection when compared to indwelling transurethral catheterization for over five days at an odds ratio of 0.142 (95% CI 0.073-0.0276).^3^ Another alternative to bladder drainage in the intubated IPP patient is clean intermittent catherization, however this poses a unique challenge in the intubated COVID positive patient as it repeatedly exposes healthcare staff the virus-carrying patient.

When compared to indwelling transurethral catherization, suprapubic tube placement has been shown to confer a lower risk of urinary tract infection and IPP infection/erosion. This can primarily be explained by its ability to drain the bladder without creating inflammation and friction in the urethra. Therefore, we propose that any team caring for a patient with an IPP and a planned, prolonged indwelling transurethral catheterization consult urology services to have a suprapubic tube temporarily placed. This will ensure that the risk of urinary tract infection and/or IPP erosion is kept as low as possible.
